# Characterization of Industrial Coolant Fluids and Continuous Ageing Monitoring by Wireless Node—Enabled Fiber Optic Sensors

**DOI:** 10.3390/s17030568

**Published:** 2017-03-11

**Authors:** Alexandros El Sachat, Anastasia Meristoudi, Christos Markos, Andreas Sakellariou, Aggelos Papadopoulos, Serafim Katsikas, Christos Riziotis

**Affiliations:** 1 National Hellenic Research Foundation, Theoretical and Physical Chemistry Institute, Photonics for Nanoapplications Laboratory, Athens 11635, Greece; alexandros.elsachat@icn2.cat (A.E.S.); anastasia.meristoudi@gmail.com (A.M.); chmar@fotonik.dtu.dk (C.M.); 2 Catalan Institute of Nanoscience and Nanotechnology (ICN2), CSIC and the Barcelona Institute of Science and Technology, 08193 Barcelona, Spain; 3 Technical University of Denmark, Department of Photonics Engineering, DTU Fotonik, Kgs. Lyngby 2800, Denmark; 4 PRISMA Electronics S.A., Research & Development Department, Industrial area of Alexandroupolis, Alexandroupolis 68132, Greece; asakel@prisma.gr (A.S.); serafeim.katsikas@gmail.com (S.K.); 5 Kleemann S.A., Kleemann Group of Companies, Kilkis Industrial Area, Kilkis 6100, Greece; ag.papadopoulos@kleemannlifts.com

**Keywords:** ageing monitoring, photonic sensors, industrial coolants, sol-gel, optical fibers, predictive maintenance, wireless sensors

## Abstract

Environmentally robust chemical sensors for monitoring industrial processes or infrastructures are lately becoming important devices in industry. Low complexity and wireless enabled characteristics can offer the required flexibility for sensor deployment in adaptable sensing networks for continuous monitoring and management of industrial assets. Here are presented the design, development and operation of a class of low cost photonic sensors for monitoring the ageing process and the operational characteristics of coolant fluids used in an industrial heavy machinery infrastructure. The chemical, physical and spectroscopic characteristics of specific industrial-grade coolant fluids were analyzed along their entire life cycle range, and proper parameters for their efficient monitoring were identified. Based on multimode polymer or silica optical fibers, wide range (3–11) pH sensors were developed by employing sol-gel derived pH sensitive coatings. The performances of the developed sensors were characterized and compared, towards their coolants’ ageing monitoring capability, proving their efficiency in such a demanding application scenario and harsh industrial environment. The operating characteristics of this type of sensors allowed their integration in an autonomous wireless sensing node, thus enabling the future use of the demonstrated platform in wireless sensor networks for a variety of industrial and environmental monitoring applications.

## 1. Introduction

There is a growing interest on the development of new techniques for efficient infrastructure monitoring and assets maintenance management in different industrial sectors, further to support calendar-based maintenance which is now a widely used industrial standard procedure. Predictive maintenance [1,2] suggests a viable solution, recently emerged, which is based on the continuous monitoring and assessment of the real conditions and the extraction of results regarding the optimum time plan and actions for maintenance [2,3]. Implementation of such predictive maintenance systems requires efficient and reliable sensing devices that could be interconnected in a network of sensors collecting and handling the information and feeding this to a decision making system for appropriate maintenance actions [2]. Photonics technology is currently a quite favorable solution for the development of environmentally robust sensors [2] with operational safe characteristics, even in demanding explosive or flammable environments [4] and which also exhibit electromagnetic interference immunity (EMI), crucial to a number of industrial applications. Certain photonics technologies [5] mainly focused on large core optical fibers, have demonstrated some unique characteristics such as low cost, easy handling, compatibility with inexpensive transceivers, and also compatibility for easy and rapid customization [6,7]. Combining such low cost fibers functionalized with sensitive materials [8,9] is a realistic way for the development of reliable yet affordable sensing heads that could be employed as reusable or disposable sensors. Further to the above requirements there are also certain operational requirements regarding the interrogation complexity, circuitry and transceiver complexity that should be kept at a minimum level of cost. To avoid complex interrogation systems operating in the frequency domain, for example, by employing components with Bragg gratings [10], amplitude interrogation is proposed as an effective balance between accuracy and complexity. Further, overcoming the need of expensive stabilized laser sources, the use of non-coherent LED sources and inexpensive photodetectors provide solutions for sensor integration in low power consumption autonomous sensing nodes.

**Figure 1:**
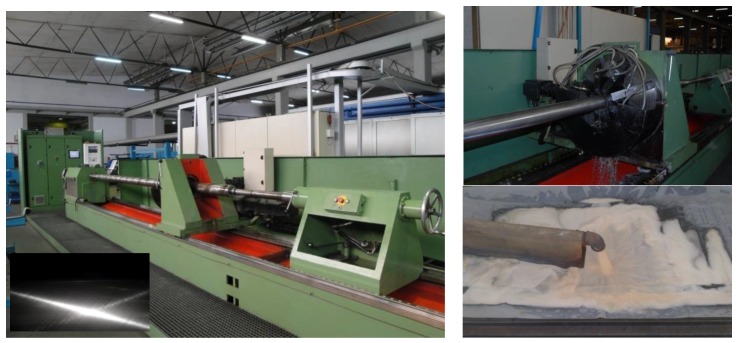
A Honnen metal polishing machine (the inset demonstrates a typical required quality of the produced metal surfaces with special line features on it.) Detail of the polishing head with the presence of coolant flow. Coolant’s exit from the grinding machine and detail of coolants’ initial filtering and transient temporary storage at the tanks.

The viability of the specific sensing platform in wireless-based systems for predictive maintenance and management is demonstrated here based on an indicative and rather challenging application of coolants fluids’ ageing monitoring [11] in a harsh industrial environment. Such autonomous and embedded sensors equipped with wireless interconnectivity capabilities [12,13,14] are expected to play a key role in the development of future Internet of Things (IoT) applications [15,16]. More specifically, the demonstration case in this work is focused on monitoring the ageing process of coolant fluids [17,18] in industrial metal processing of metallic cylinders (pistons) used in hydraulic elevators in the industrial collaborator Kleemann S.A. (Kilkis, Greece). Different machines for grinding (BOSSI s.r.l, Milano, Italy) and for polishing with custom made Honnen machine (Kleemann S.A., Kilkis, Greece) are employed for metal processing to strict manufacturing requirements (Figure 1). For the dissipation of the heat and the maintenance of constant lubricating action the use of coolant fluids is essential [19]. Depending on the coolant type there are specific conditions for their proper operation in order to provide the desired cooling and lubricating actions together with anticorrosion and antibacterial protection [18]. Failure to maintain proper cooling action can lead to significant deterioration of metal processing quality, leading in turn to damaged metallic parts or broken machine tools, with the corresponding time delays in the production line and the associated often very serious economic costs.

Additionally, during the metal grinding process, the change of coolant’s characteristics can lead to development of bacterial or microbial biohazardous load [20] where at a certain level of ageing becomes non-usable, due also to serious intolerance by the personnel and other associated health safety issues [21]. Despite the fact that there are no systematic studies in the literature for quantifying the coolants’ ageing process or providing continuous monitoring strategies, there is in contrast a large number of studies on the toxicity and health effects of coolants associated with skin irritation and respiratory effects to even more severe pulmonary complications [21,22]. Therefore the continuous monitoring of coolants is very important not only for guaranteeing end-manufacturing specifications but also for meeting the strict environmental and health issues regulations. Furthermore at the end of a coolants life-cycle when it becomes unusable there arises in coolants’ management process the issue of their proper disposal and replacement. This is also directly related to environmental and economic issues associated with optimized fluid waste handling policies [23] and efficient storage and logistic processes. Additionally the storage tanks of pristine coolants need also to be monitored as they are highly susceptible to deterioration due to potential development of bacterial load under certain environmental storage conditions of temperature, humidity or atmospheric pollutants [22]. The presence of different metal processing machines in the same large scale infrastructure together with remote sites of storage and waste coolant tanks create the need of a coordinated monitoring by wireless sensor networks for providing the overall combined condition of coolants and allowing the efficient management by a decision making system.

In terms now of the actual sensing strategy the key point is to identify if possible a simple parameter approach in order to monitor the condition of such chemically complex multi-component coolant fluids. As will be shown later the acidity (pH) can be related very accurately to the condition of the coolant. However the operation of a sensor in direct contact with coolant fluids has not been systematically evaluated before and there is no prior information on the actual response of similar sensors. The behavior of such sensors is expected to be much different in real coolant fluids as compared to standard solutions of controllable pH. Different large core optical fiber sensors were characterized in coolants monitoring and successful implementations were identified here as robust enough in terms of lifetime and repeatability.

Preliminary results of coolants characterization and sensors behavior were previously reported [11], while here a thorough analysis leading to the optimization of the sensors response that enabled also their integration in low power driving circuits boards is presented. By properly selecting low power optical transceivers it was possible the integration of fiber optic sensor in an autonomous wireless sensing node in a ZigBee wireless sensor network. This functionality can enable the efficient monitoring and management of the entire cycle of coolants’ lifetime, from storage, to operation and finally to the disposal phase, in a large scale industrial infrastructure.

The paper is organized in the following way: first the detailed chemical, physical and spectroscopic characterization of the coolants aging process is presented in order to reveal the ageing mechanism and identify the suitable parameter for efficient monitoring, thus allowing the design of appropriate sensing components. Then is presented the fiber optic sensors development by specially designed coating of sol-gel derived pH sensitive materials, followed by the characterization of sensors’ performance in standard ideal solutions and in real coolant fluids for a large range of ageing grade and associated pH index. Finally the circuitry integration of the sensors in a wireless sensing node is discussed and demonstrated.

## 2. Materials and Methods: Coolants Characterization and Sensing Approach

### 2.1. Coolant Fluids’ Study and Characterization

#### 2.1.1. Industrial Coolant and Lubricant Fluids

The study has been based on two representative and commercially available coolant fluids widely used in industry. Specifically, those coolants were employed in metal grinding and polishing machinery in the infrastructure of the industrial partner Kleemann S.A., providing thus access to real working conditions data and allowing the detailed characterization of coolants aging process. The two specific coolant liquids used are Syntilo 81 BF by Castrol [24] and Multan 61-3 DF by Henkel [25].

Syntilo 81 BF is a yellow colored coolant liquid that presents a high level of viscosity. It is mainly used in heavy industrial machinery in an 8% diluted form. The main ingredient of Syntilo 81BF is triethanolamine (35%–40%) in addition to several other ingredients such as ethanolamine (1%–5%), 3-iodo-2-propynylbutyl carbamate (0.1%–1%) as well as poly(quaternary ammonium chloride) (0.1%–0.25%). Table 1 summarizes the chemical composition of Syntilo 81 BF coolant liquid which is commercially available from Castrol. The main ingredients of Multan 61-3 DF by Henkel (Table 1) are polyalkylene glycol esters; however, the exact amount (%) of this particular material is not provided in the Henkel specifications. Similarly, this coolant liquid contains a small amount (0.25%–0.3%) of pyridine-2-thiol 1-oxide sodium salt, as well as several inorganic salts.

Deterioration of coolants in metal grinding applications [18,19] can have several implications in the effectiveness of their operation as well as in the environmental conditions [20,21,22,23], and more specifically could result in the presence of rust on the metal parts, premature tool wear due to lack of lubricant in the coolant, excessive split emulsion that could induce particle filter blockages, as well as excessive skin sensitivity and foul odors due to emissions of sulfuric and hydrochloric acid. The ageing of the coolant can be attributed partially to the elevated operating temperature in the working parts of the machines (35–50 °C) and also to the metallic impurities trapped inside the liquids, after the long friction with the metal pipes. Based on the temperature measurements performed during the grinding process the temperature range was between 35 and 50 °C at the friction/grinding area. The coolant fluid exits the machine at a temperature between 32 and 35 °C and afterwards is stored at room temperature at the adjacent tank (Figure 1). The temperature at the friction area is estimated to be up to 60 °C. An efficient and continuous monitoring of coolants acidity along their life cycle could provide an efficient tool for their operation and could also to reveal useful trends that would allow the on-time predictive actions for coolant replacement.

#### 2.1.2. pH Characterization

**Figure 2:**
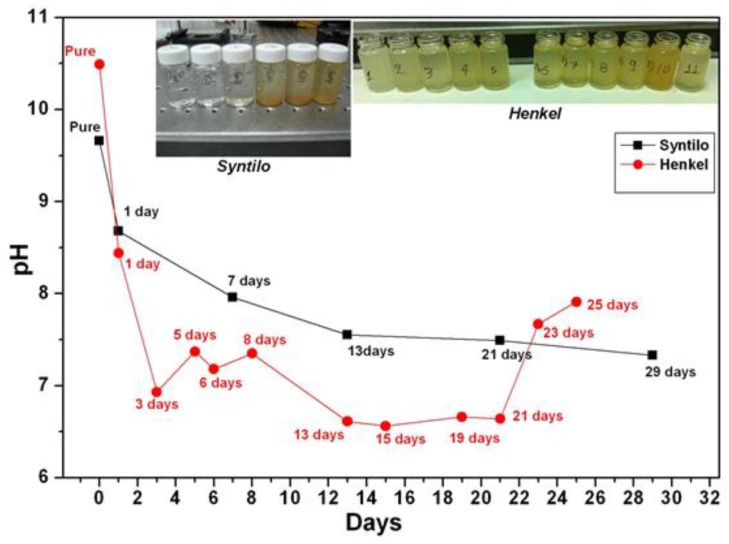
pH characterization of Castrol and Henkel coolant fluids at different degrees of ageing (days used).

Coolants are designed to be alkaline in order to neutralize the acidic nature of the developed biological content (e.g., bacteria) entering and growing in the coolant fluid tank. Empirically the maintenance and the management of the coolant fluid tanks is addressed by manually measuring its acidity. Here a detailed study of pH monitoring of coolants was performed at different stages of aging in order to reveal the dependence of pH with coolants’ aging. The coolants were sampled during the operation of the grinding machine, collected in bottles and measured afterwards in the lab environment using a high resolution commercial electro-chemical pH meter (PIONEER, Radiometer Analytical SAS, Lyon, France). Figure 2 summarizes the measured values of pH for the Syntilo 81 BF by Castrol (black line) and Multan 61-3 DF by Henkel (red line). Considering the first coolant liquid by Castrol, it is clearly observed that as the coolant’s use duration increases, the corresponding pH index are decreasing monotonically. These variations range from 9.6 to 7.3 during a 29 days use period. Similar behavior was observed in the second coolant liquid, provided by Henkel. However, the pH values of Multan 61-3 DF are lower compared to Syntilo 81 BF. The range of the second liquid’s pH index is from 10.5 to 6.6 over the 25 days period of use.

Due to specific operational requirements of the production line during its operation, fresh coolant was added when this was empirically judged by the personnel as necessary due to the anticipated increased need of cooling and lubricating action. This is usually an empirical action based on the visual inspection of metal pipes for grinding and depends on their surface quality characteristics like roughness or cleanness, as this determines the required grinding strength. The addition of fresh coolant in the tank took place as demonstrated in the days numbered as 5, 6, 8, 23 and 25 and indicated with the irregular pH values not following the monotonically decreasing trend. This set of data was intentionally included in the study, regardless of the associated interpretation problem caused by the addition of the coolant that had to be done according to the current rules of the factory production line, as it demonstrates characteristically a common current coolant management approach. This empirical decision and followed management process often leads to an over- or underestimation of the real situation with the obvious associated inefficiency of the maintenance strategy.

During the operation of the grinding machines the coolants are recirculated and stored in tanks after passing through suitable microporous textile filters (Figure 1) in order to remove residues. The collected samples are filtered by this standard way but a further filtration step would be possible if required, in order to assure the minimum level of particle contaminants and residues. This would help the monitoring process of the coolants by the sensing element leading possibly to longer lifetime of the sensors.

**Figure 3:**
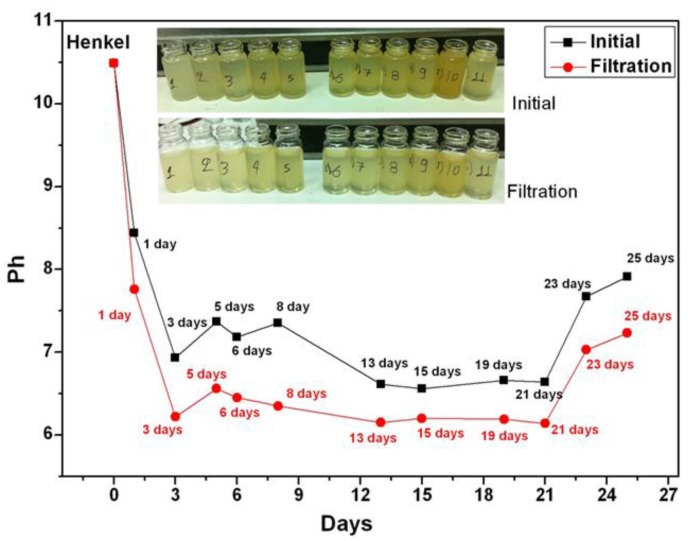
pH measurement before (black) and after (red) the additional coolants’ filtration for the Henkel Multan 61-3DF. A clear reduction of the pH index for the filtered coolant is noticeable.

Collected coolant samples were filtered by microporous paper-type filter while examining how this process affects the pH value of the samples. The comparison of the pH values of the samples before and after filtration is shown in Figure 3, where it can be noticed that after the filtration the aged coolants’ pH values decreased by almost a full pH unit. However, the overall pH trend of the aged coolant remains the same, characterized by a monotonically decreasing trend.

#### 2.1.3. Spectroscopic Characterization

**Figure 4:**
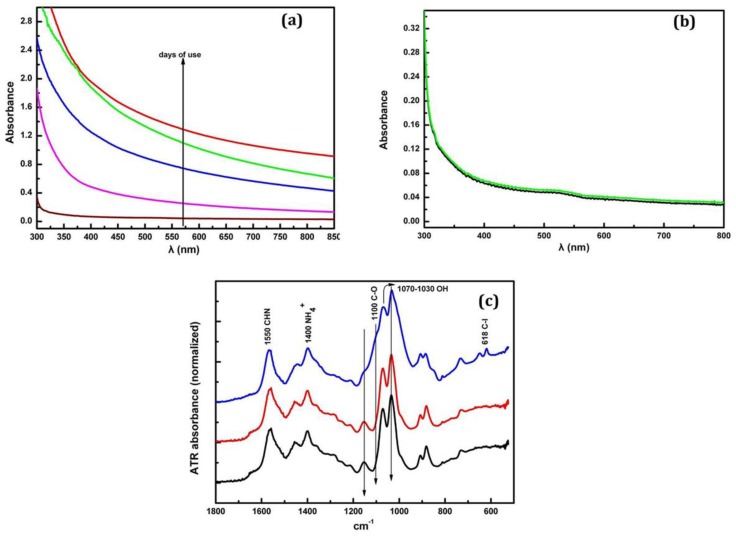
(**a**) Absorption spectrum of Castrol Syntilo 81 BF coolant over 28 days of use (**b**) comparison of the pure material (black) and after 28 days (green) absorption spectra and (**c**) ATR spectrum of coolant before use (black), after 7 days (red) and 21 days (blue) of use.

In order to study the properties of the coolant fluids and also monitor their behavior during the aging period a full spectroscopic study was performed employing spectroscopic techniques such as UV-Vis absorption and Attenuated Total Reflectance (ATR) spectroscopy. The spectroscopic characteristics of the coolant materials were monitored during the aging process while employed in the metal grinding machines over a typical period of 28 days. For the study samples of aged coolants were collected at the first day of operation and afterwards every 7 days. Figure 4a shows the absorption spectrum (UV-Vis) from 300 to 850 nm wavelength of Castrol Syntilo 81BF, where a clear shift of the entire absorption spectrum to higher values with respect to the days of use is observed. This change can be attributed to the increase of scattering phenomena due to the presence of metallic impurities in the sample during the evolution of the grinding process. Figure 4b provides as reference the absorption spectrum of the unused pristine coolant sample showing that it remains identical after 28 days regardless of the storage conditions. In order to further investigate the material’s ageing process and operational degradation caused by its use, infrared ATR spectroscopy over the range 650 cm^−1^ and 1800 cm^−1^ was applied. Figure 4c presents the peak characteristics introduced by the group vibrations of the material ingredients. In particular, at 1550 cm^−1^, it is clearly observed the vibration of the C-H-N group due to the existence of amines in the solution.

The vibration of NH_4_^+^ occurs at 1400 cm^−1^ and similarly at ~620 cm^−1^, the vibration of C–I groups, due to the presence of 3-iodo-2-propynylbutyl carbamate. All the other vibrational peaks appearing in the ATR spectrum could possibly be caused by the impurities introduced in the coolant liquid during its operation. Furthermore, a relative intensity variation of the peaks in the range of 1030 cm^−1^ and 1100 cm^−1^ which is the vibrational region of –OH and C–O groups can be clearly observed. This intensity variation probably occurs due to protonation and deprotonation caused by the presence of impurities in the liquid sample. Following the same procedure all the aforementioned spectroscopic techniques are repeated in order to study the second coolant fluid (Henkel Multan 61-3 DF).

**Figure 5:**
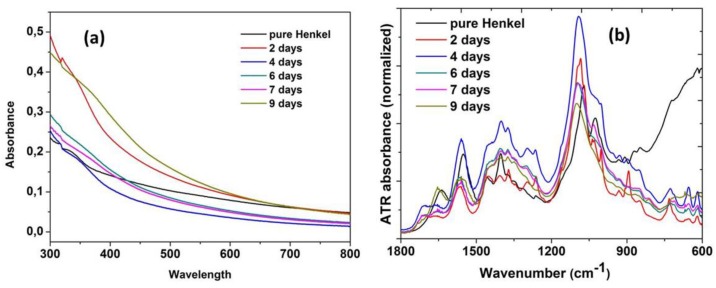
Spectroscopic characterization of coolant Henkel Multan 61-3 DF (**a**) absorption; and (**b**) ATR spectrum, for a 9 day ageing interval.

Figure 5a shows the absorption spectrum (UV-Vis) from 300 to 800 nm, and Figure 5b shows the ATR absorbance spectrum trend over 9 days. The irregular behavior is due to the additional coolant liquid added in the machine, thus changing the chemical composition of the samples as described in the previous section.

### 2.2. Fiber Optics Sensor Design and Development

The previous analysis of the two coolant fluids’ ageing mechanism, has allowed the determination of their chemical and physical parameters in connection to their ageing mechanisms and the identification of suitable monitoring parameters. Due to the nature of the coolants and the apparent residues and strong presence of biological content such as bacteria grown during their operation, special care should be taken in order to either protect a rather expensive measuring device or employ a low cost and disposable sensing head that would be able to withstand for a reasonable and adequate time of operation in a reliable way. Given that the coolants fluids are actually a harsh environment for monitoring, the use of conventional pH meters suggests a problem with the lifetime of their employed electrodes as they are immersed on those liquids [26]. On the other hand, optical fiber-based pH sensors, compared with the corresponding electrical pH sensors, exhibit many advantages such as compact size, electrically passive operation, immunity to electromagnetic interference, capability of remote measurement and multiplexed detections [27,28]. Several fiber-optic pH sensors have been demonstrated to date by depositing a pH-sensitive coating onto the fiber surface such as hydrogels [29], cellulosic films [30], sol-gel derived glassy materials [31,32].

Recently new methods have been proposed which are based on layer-by-layer (LbL) electrostatic self-assembly (ESA) techniques [33,34] combined with the utilization also of special structures like nanostructure cavities [35], long period gratings LPG [36] or tilted fiber Bragg gratings [37] but with a limited pH range monitoring capability. However in order to provide a platform with the desired operational characteristics combined with low cost components, low power consumption, ease of handling, low cost processing, and environmental robustness in terms of electromagnetic interference EMI, the large core optical fibers [8,9] with appropriate sensitive overlayers offer a well-balanced solution. Therefore, polymer and silica optical fibers were considered and functionalized here using the fabrication flexibility of the sol-gel derived materials [38] by the appropriate incorporation of suitable pH indicators towards the development of pH sensitive overlayers.

The experiments were performed with both polymer and silica fibers in order to demonstrate a direct comparison of their performance in terms of pH variation in standard solutions and in industrial coolant liquids respectively. Silica fiber of total diameter 630 μm was purchased from Thorlabs GmbH (Munich, Germany) and the polymer fiber used was an ESKA GH-4001P of 1 mm total diameter, manufactured by Mitsubishi-Rayon Co. (Tokyo, Japan). To retain the simplicity of the sensing device the sensor measuring principle was based on an amplitude interrogation system that is the simplest possible, but also adequate for its purpose in this demonstrated application. The operation of the proposed sensing device is based on evanescent wave absorption (EWA) phenomena [39,40]. These sensors are typically used to monitor the concentration of an absorbing material allowing the direct interaction with it. Evanescent waves in the cladding region were exploited in order to develop sensors based on evanescent wave absorption mechanism [41,42]. Evanescent field absorption occurs when the medium, which forms the cladding of the waveguide, absorbs the light at the wavelength being transmitted through the fiber. In our case the intermediate reagent which will interact optically with the coolants is the sol-gel based fiber’s overlayer. Three different pH indicators were used in order to extent the response to the required detection pH range of coolants. The aforementioned pH indicators were activated under specific pH conditions causing alterations in absorbance measurements at a specific wavelength, thus affecting the transmitted guided light. The quality and the efficient deposition of the sol-gel layer on the fiber surface, the proper functionalization of the fiber surface before the deposition and the proper selection of the pH indicators are critical parameters to the development of the fiber sensor.

#### 2.2.1. Sol-Gel Overlayers Synthesis

The chemical reagents and the materials used for the synthesis of the active sol-gel derived sensing material were tetraethylorthosilicate (TEOS) combined with cresol red, bromophenol blue and chorophenol red indicators which were all obtained from Aldrich Chemical Co (Milwaukee, WI, USA). In order to adjust the pH to the desired value, suitable quantities of 0.1 mol/L hydrochloric acid (HCl) and 0.1 mL/L sodium hydroxide (NaOH) solutions were used.The synthesis of the active material was conducted at ambient conditions by mixing 3 mL of TEOS with 1 mL deionized water, 3 mL of ethanol, along with 4.6 mg cresol red, 8.2 mg bromophenol blue and 4.2 mg chlorophenol red. Finally, a 3% solution of Triton X-100 was added to the material solution in order to act as a surface active reagent and significantly reduce the micro-cracks in the formed sol-gel derived film. The solution was let to stir for 60 min at 60 °C, in order to allow the polymerization and the network formation of the silica matrix, which incorporates the pH indicators. Table 2 shows the characteristics of the pH indicators used in our experiments having dynamic pH range (3.0–10.8).

#### 2.2.2. Fiber Surface Preparation

Two different type of fibers were considered and studied in this work, a silica (SiO_2_) optical fiber with core diameter equal to 600 μm and total diameter 630 μm and also a polymer fiber (PMMA) with core diameter equal to 980 μm. Both fibers have a removable cladding, a 15 μm thickness polymer cladding for the silica fiber, and a 10 μm thickness fluorinated polymer cladding, for the polymer fiber. For the preparation of the fiber sensing region, the cladding of the fibers was initially removed using acetone. Thereafter, both the uncladded silica and polymer surfaces have been activated by using a 30% HNO_3_ solution, in order to assist the bonding of the glassy sol-gel film with the surface of the fibers. Tetraethylorthosilicate (TEOS) was used as the main liquid precursor for preparing the pure silica film on the unclad fiber, whereas the silica glass gives a refractive index less than that of the fiber core and consequently, allows the total internal reflection guiding condition to be satisfied. Generally, TEOS forms a three-dimensional network of silicon dioxide (SiO_2_) through hydrolysis and polymerization process and pH indicators can be trapped in structure without any degradation or leaching. The material deposition to the glass and polymer surface of the fibers was achieved in a controllable manner by immersing the fibers (dip-coating method) into the sol-gel solutions for a fixed amount of time with successive repeats. After the deposition/coating, the fiber was left in a fume hood at atmospheric pressure and room temperature for ~20 days. Finally, the fibers were annealed at 60 °C for 24 h to remove any remaining organic compounds and hydroxyl residues and were also washed with deionized water to remove the unbound indicators.

#### 2.2.3. Experimental Set-Up

The characterization of the optical and electrical response of the developed fiber optic sensors was performed by using a low-cost LED source operating at 650 nm with maximum output power of 1 mW, (IF-E99, Industrial Fiber Optics, Tempe, AZ, USA).

**Figure 6:**
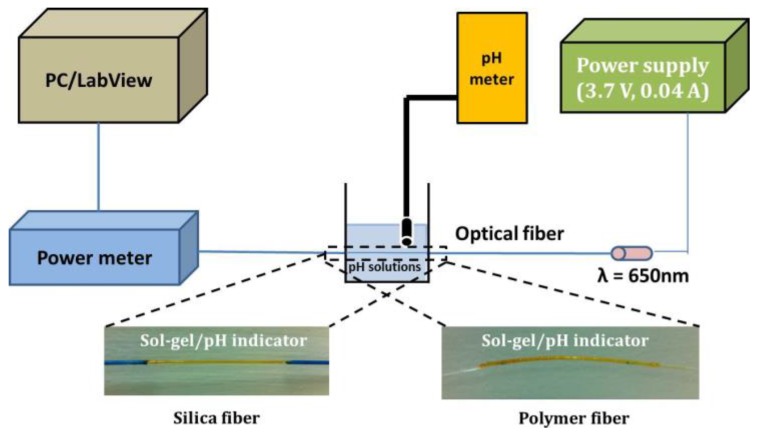
Schematic of the experimental set up with indicative photos from both silica and polymer optical fiber sensing heads after the deposition of the sol-gel derived pH sensitive overlayer.

The light was coupled into the fibers and collected by an optical power meter (model 2832-C Dual Channel, Newport Corporation, Irvine, CA, USA) connected to a PC. The measured optical signal was continuously recorded over time using custom-built software based on LabView. The characterization of the electrical response was performed also with a low cost photodarlington detector (IF-D93, Industrial Fiber Optics) with integrated high optical gain and linear response. Both the photodiode and phototransistor have integrated micro-lenses for efficient coupling to standard 1000 μm core optical fiber facilitating the easy connection with the fibers. The sensors were characterized both in standard solutions with predetermined acidity where the pH indices were achieved by adding HCl or NaOH solutions, and the resulted value was also confirmed measuring it with a high resolution commercial electro-chemical pH meter (PIONEER). Figure 6 shows the employed experimental set-up in order to perform the optical measurements and characterization of the fiber sensors. The active sensing region of the fibers (silica and polymer) is illustrated below. The same experimental set-up was used for both silica and polymer fiber sensor characterization.

The specific LED source at 650 nm was selected taking into account the optimised wavelength for operation of the specific pH indicators mixture that has been previously identified to be around 620 nm [41]. The selected source was the best compromise given that at the time of sensors’ design and development it was the most powerful commercially available compact LED source with low power consumption.

## 3. Results and Discussion

This section focuses on the performance characterization of the developed fiber optic sensors. Polymer- and silica fiber-based sensors were tested initially in standard pH solutions in order to validate their performance and provide a reference response, and afterwards in the selected samples of aged coolant fluids. The temperature of the liquid samples during the experiments was kept at 21 °C.

### 3.1. Sensors’ Characterization in Standard Solutions

**Figure 7:**
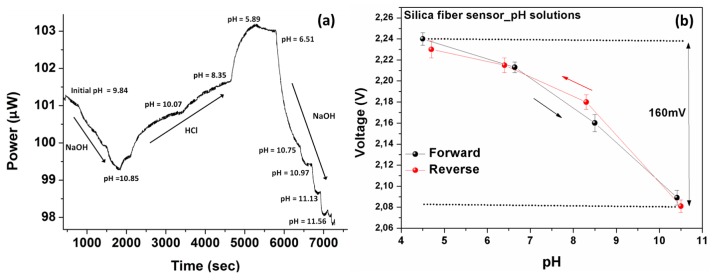
(**a**) Measured optical response of the silica fiber sensor standard pH solutions following dynamic changes in solutions’ acidity. The dynamic response of the sensor in successive solution’s acidity changes is clearly demonstrated. (**b**) The electrically converted response of the silica optical fiber sensor, as a function of the pH, exhibiting an almost linear and also reversible response with an adequate 160 mV range.

Experiments have been carried out for the performance evaluation of the developed fiber optical sensors for both silica and polymer material platforms. The diagrams in Figure 7 shows the effect of solutions’ pH index variations on the transmitted optical power in the case of silica fiber. The variation of the pH levels of the under study solution achieved by adding HCl or NaOH solutions in different (random) time points respectively during the experimental procedure. During the sensor characterization the acidity of the solutions was also measured with an electrochemical pH meter. As illustrated in Figure 7a, successive changes of solution’s pH index produces corresponding changes to the optical power of the optical fiber sensor with very good dynamic response and reversible characteristics.

The electrical characteristics of silica fiber-based sensor’s response was also measured as a resistor after the phototransistor converted the current response into a voltage. The measured electric signal range was 2.09 V to 2.24 V (without using an amplifying circuit) for the entire monitored pH range, as shown in Figure 7b. The sensor exhibited an overall optical response of about 5.2% in the (10.5–4.5) pH range. The repeatability of the electrical response was evaluated as well, and is also shown in Figure 7b along a full cycle of a pH changes, demonstrating very consistent results for such a wide pH range. The range of the electrical response in the whole measured pH index region was equal to 160 mV. This voltage range is evaluated as compatible with digital board circuitry systems that can allow the integration of sensors in wireless sensing nodes with appropriate interrogation systems.

**Figure 8:**
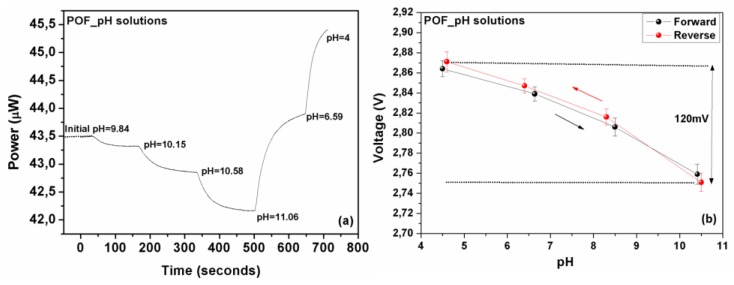
(**a**) Measured optical response of the polymer optical fiber sensor standard pH solutions following closely dynamic changes in solutions’ acidity. The dynamic response of the sensors in a successive solution’s acidity changes is clearly demonstrated. (**b**) The electrically converted response of the polymer optical fiber sensor, as a function of the pH, exhibiting an almost linear and also reversible response with an adequate 120 mV range.

Further to silica-based optical fiber sensors, standard PMMA polymer optical fiber-based sensors were also characterized. Following the same characterization method as above the sensor was evaluated and the results are presented in Figure 8a, showing the variations of the output optical power for a range of solution pH indexes. As illustrated the behavior of the polymeric optical fiber sensor seems similar to that of the silica fiber in the case of pH standard solutions exhibiting monotonous linear responses. The polymer optical fiber sensor showed an overall optical response of about 7.5% in the (10.5–4.5) pH range while the corresponding electrical signal increased from 2.75 V to 2.87 V, as shown in Figure 8a,b, respectively.

The range of the electric voltage for the whole region of the pH index variation is measured to be 120 mV, with very good repeatability.

### 3.2. Sensors’ Response in Coolant Fluids

The initial characterization of sensors in ideal standard solutions with predetermined pH values, was important in order to assess their capabilities and verify their effectiveness. However, the testing in ideal pristine solutions [18] is far from the real aged coolant fluids situation we are dealing with in this study, which have a quite complicated chemical composition [20,22]. More importantly coolant fluids develop during their aging progresses a quite heavy and complex biological load (e.g., bacteria) and incorporate also a number of residues and pollutants from the ferrous materials and the surrounding industrial environment present even after the filtering process. Therefore, the coolant fluid system could be characterized as a rather harsh environment for monitoring. This would definitely affect the measuring needs and also affect strongly the lifetime of the monitoring sensors. Indeed, although the first evaluation of the sensors’ performance showed similar results for polymer and silica optical fiber platforms, their behavior in coolant liquids is quite different as presented below.

**Figure 9:**
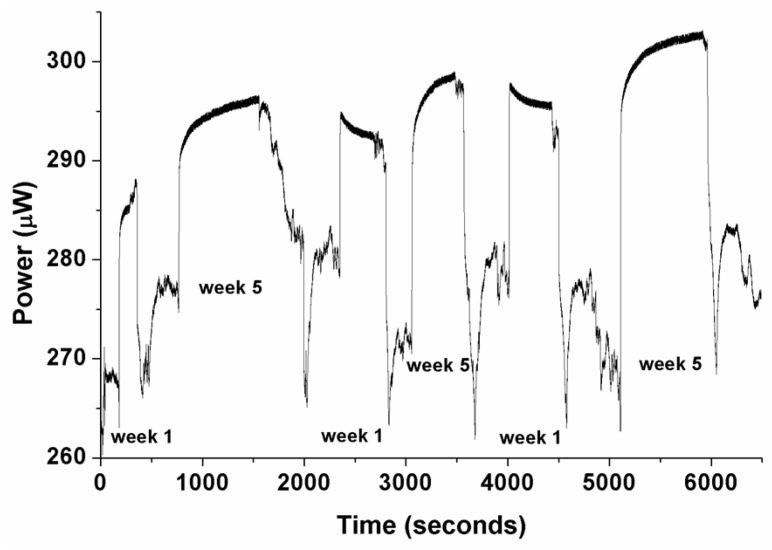
Measured response of polymer optical fiber sensor in two different samples of coolant fluid Castrol Syntilo 81BF with two different aging degrees (Week 1 and Week 5), after successive measurements. The inconsistency of the sensor’s behavior in real coolants, due probably to its deterioration in response to attack by the coolant’s specific components is clearly demonstrated.

Figure 9 shows the optical response of the polymer fiber sensor in two different coolant samples of different aging (Week 1 and Week 5) of Syntilo 81BF, where the optical response appeared to be unstable and inconsistent after successive measurements. This could be attributed to the gradual deterioration of the overlayer’s quality in the presence of the attacking coolant fluid. In general, it is anticipated that due to the fact that the glassy nature of the sol-gel originated overlayer has different mechanical characteristics (e.g., expansion and thermal coefficients) compared to the polymer fiber surface would be more susceptible to cracks. This will result in partial delamination of the overlayer and the overall deterioration of the sensor.

In contrast, the better deposition quality of the sol-gel derived material on the silica modified fiber substrate, led to the development of a stable sensitive layer strongly bonded to the silica substrate thus capable to withstand the presence of coolants and other impurities and allow its operation to determine the pH in this harsh environment.

**Figure 10:**
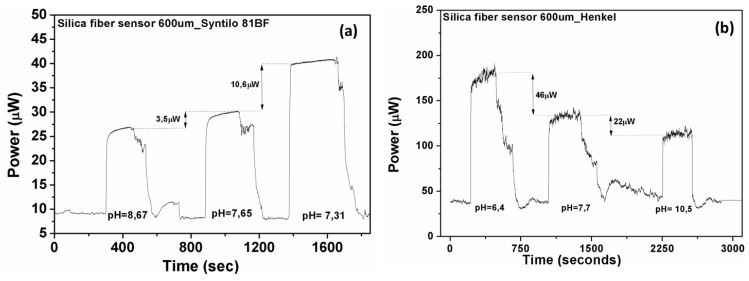
Measured optical response of silica optical fiber based sensor in both coolants (**a**) Castrol Syntilo and (**b**) Henkel Multan.

Figure 10a,b shows the different optical responses of the silica fiber sensor in the two different coolant fluids. The time response of the sensing heads is almost instantaneous. For the cases of monitoring the pH range (7.3–8.6) for the Syntilo and (6.4–10.5) for Multan samples, the total reduction in percent of the optical signal was calculated based on the experimental results and found equal to 34% and 38%, respectively. The pH sensor is capable of determining the critical points of coolant deterioration during the ageing process.

**Figure 11:**
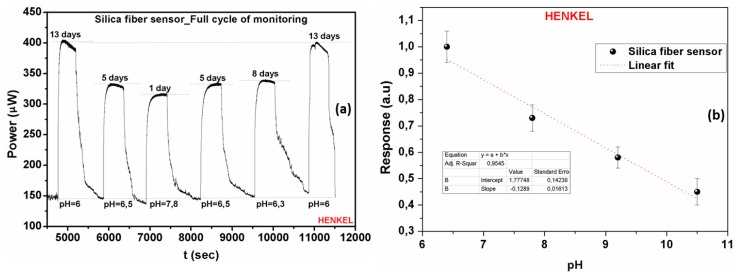
(**a**) Experimental evaluation of the silica optical fiber-based sensors’ response and its exhibited reversibility during monitoring of the Henkel coolant ageing process. The fast time response to the coolants’ presence is also demonstrated. (**b**) Normalized electrical response of the silica optical fiber sensor as a function of the pH index in the Henkel coolant fluid, for the entire pH range of coolant operation.

Based on the availability of coolant fluids, another set of measurements was performed in order to assess the reversibility of the employed sensors along a full cycle coolants’ use. Figure 11a presents the optical response together with the time response characteristics of the silica fiber sensor during a full ageing cycle experiment of the Henkel Multan coolant. Following a number of additional sensor characterization measurements the electrical response was also derived for the entire working pH range of the Henkel coolant (Figure 11b). The specific pH range was chosen taking into consideration the different ageing intervals of the coolant liquids, as already shown in Figure 2. Based on this fact, the determination and prediction of their full life cycle can be achieved from the exhibited monotonic linear behavior.

The experimental procedure for the sensor characterization lasted in the last demonstration example more than three hours and during this period the sensor was able to accurately monitor in real time the pH index of coolants. Similar behavior in time extended characterization was observed in other coolants and in standard pH solutions. This continuous contact of the sensing heads with the coolants could have an impact to the expected lifetime of the sensor, but deterioration is not obvious in the demonstrated examples. Given the relatively slow change of the coolants’ characteristics during the real monitoring process, the coolants can be sampled and assessed for only a few seconds with a typical sampling period of 1 to 2 h, therefore the operational lifetime of the sensor could readily exceed one month of operation which is more than enough as the coolant is typically almost fully replaced in three weeks. The outcome of this procedure demonstrates the efficient and reversible operation of the proposed inexpensive sensors under harsh conditions, making them viable for industrial applications. Of course for real applications certain improvements of the sensors are needed for efficient standardization that would enable the easy replacement of the sensing head which is in any case of very low cost. We also have to note that due to the nature of aged coolants, the developed biological load increases considerably, even under normal laboratory storage conditions even without any presence of metal processing. Therefore it was not possible to directly measure and compare with the same sensing heads the previously examined coolants as after a few days/weeks period their composition had changed. However we have examined the response of the sensing heads in standard solutions after 5 and 10 days following their use for coolant characterization and confirmed a very good and acceptable repeatability.

### 3.3. Sensor Integration in Wireless Node Modules

**Figure 12:**
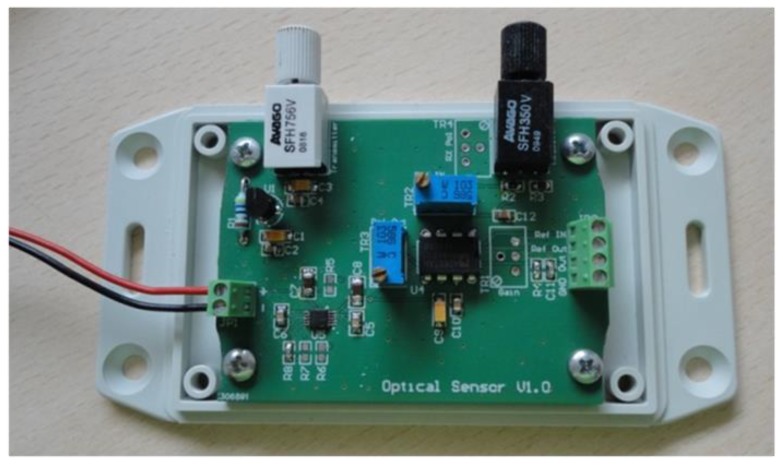
Prototype development of the Fiber Optic Driving Circuit Board. Integration of the LED source and the photodetector in the same battery operated PCB-board.

The objective of this study was to identify viable low cost and low complexity sensing platforms that could be integrated efficiently in autonomous sensing units capable of forming a distributed monitoring network. The development of such sensors for a challenging monitoring application like coolant aging demonstrates the expected applicability of the approach to other also sensing problems, e.g., for gas sensing in large scale environmental applications. The selection of large core optical fibers allows the use of low power light sources and photodetectors that could be integrated in the sensing unit with low power requirements. Initially a lab prototype of a Fiber Optic Driver Circuit Board (FODCB) [7] supplied by 2 AA batteries (3 V in total), for the integration of the LED source (1000 microwatts output optical power at 650 nm) and a phototransistor was developed, as shown in Figure 12.

**Figure 13:**
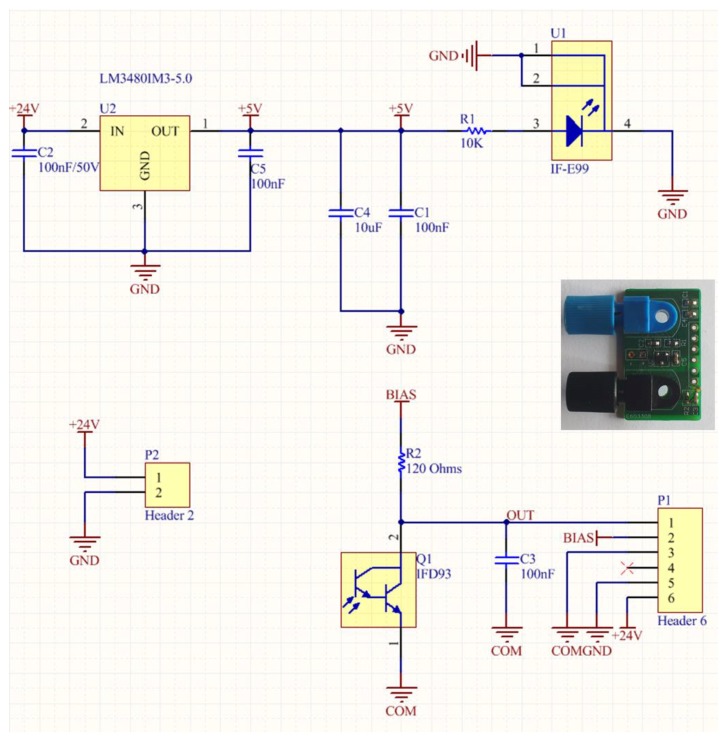
Schematic of the compact optical interface for the integration of the optical transceiver module in the wireless ZigBee unit. Inset: Photograph of the developed PCB with the attached transceivers.

The driving circuit board and the interface of the optical sensor were further improved and optimized in terms of footprint dimensions in order to be accommodated in an environmentally robust wireless node unit. A new low cost PCB was designed, as can be seen in the inset photograph of Figure 13, in order to accommodate inside the node module of the LED source (IF-E99, Industrial Fiber Optics 650 nm with maximum output power of 1 mW) and the photodetector (Photodarlington configuration IF-D93, Industrial Fiber Optics). The chosen photodetector has an integrated high optical gain and performs linearly over a broad range of temperature. The PCB hosting the LED source and the photodetector utilizes two separate voltage rails (Figure 13). 5 V is used to power the LED and 3.3 V is used to bias the output of the photodetector. The LED is fed through a 10 KOhm resistor resulting in 2.5 mW of consumption, while the photodetector is biased through a 120 Ohm (R2) resistor adding another 90.75 mW power. The total power drain on the optical board is 93.25 mW. The amount of dissipated power is fairly small and would not impact the autonomy of a battery powered device. In order to avoid a noisy power supply, which could degrade the accuracy of the optical transceiver, a low noise Low Dropout (LDO) DC-DC regulator from Texas Instruments (Dallas, TX, USA) was chosen. The LM3480IM3 can exhibit almost 70 dB of Power Supply Rejection Ratio (PSRR) over a broad range of frequencies, resulting in a stable output with minimal voltage ripple. This IC is used only for the 5 V rail, while the rest of the system is operating at the 3.3 V level. This way, the analog output of the photodetector can be directly digitized and processed in the microcontroller unit (MCU) of the wireless node, without the need of additional interface circuitry.

**Figure 14:**
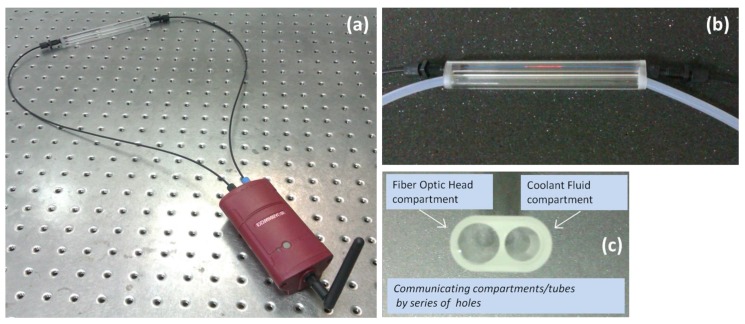
(**a**) Measuring apparatus with the sensing head connected to the wireless sensing node unit; (**b**) Photograph of the glass measuring cell with the two interconnected cylindrical inner compartments for the accommodation of the fiber optic sensing head and the coolant flow system; (**c**) Photograph showing in detail the dual tube glass cell.

The wireless node is hosted in an IP54 environmentally protected enclosure (Figure 14a) and transmits the sensors data through a ZigBee network in the 2.4 GHz by using a ZigBee to WiFi Gateway wireless communication unit [43]. All the components, including the power supply circuits, reside inside the device allowing this way an easy and reliable installation. The sensing head could be immersed directly in the coolant, however in order to avoid any potential damage by increased laminar or turbulent flow inside the coolant tank that could potentially degrade the mechanical robustness of the sensor, another scheme was developed and employed. A custom made solid glass enclosure with a dual communicating tube system was used. The sensing head was inserted and centered in the first inner tube where at the two ends of the tube the two associated fiber connectors were safely and hermetically attached. The sensing head was connected to the wireless unit with two POF extensions, as seen in the Figure 14a. The second tube, as can be seen more clearly in Figure 14b,c, was used to control the flow of the coolant fluid in the sensing head’s compartment. The coolant fluid was directed to the glass enclosure by a pump in a controllable manner, where after the successful assessment of the coolant and the corresponding reading of the sensor, the pump emptied the entire coolant content from the glass unit. This way any sudden and shocking contact of the fiber head with the coolant was avoided and also it was minimized the total immersion time of the head in the coolant during the sample monitoring procedure.

A number of issues could be addressed fοr the further enhancement of sensors’ response in this demanding application. Optimizing the deposition conditions of the sensitive overlayer and also the quantity of Triton X-100 added, could further improve the overlayers’ uniformity and reduce the micro-crack formation in the deposited sol-gel derived film, especially in the case of polymer optical fiber sensors, thus improving their robustness and reusability. Furthermore, the sensors’ response can be improved by engineering the fiber optical sensitivity by the well-known diameter tapering technique [44] that enhances the evanescent field at the fibers’ surface and consequently the interaction strength with the overlayer and the measurands.

An alternative approach for coolant monitoring could be also possibly considered based on their intrinsically different absorbance for different degrees of ageing, as indicated in Figure 4a and Figure 5a. The approach of directly sensing their absorbance properties by an evanescent wave mechanism [45] using suitably modified fibers with simply removed cladding and not by immersing the pH sensitive overlayers is probably a different approach to the one employed here. By selecting the optimum interrogation wavelength that corresponds to the highest absorbance and the better discrimination of differently aged coolants a simplified approach could be possible, although the expected linearity and responsivity would be much lower and would also be greatly dependent on the specific coolants’ composition (or other residues and absorbing biological load), thus limiting the applicability and flexibility of the sensor in real applications. In a further analysis the different absorbance of coolants at the operating light employed here in the pH responsive overlayer approach, is expected to have minimal or zero effect as the evanescent tail of a fiber’s field cannot overlap directly with the coolant given that it cannot penetrate further than the entire thickness of the deposited overlayer.

The optimization of the wireless sensing unit and its detailed systemic operational characterization in a wide wireless sensor network that monitors multiple parameters in the operation of the entire coolant fluid management and machinery maintenance in a large scale infrastructure system, is currently in progress and will be reported elsewhere.

## 4. Conclusions

For the successful penetration of predictive maintenance techniques in the management of infrastructures and industrial assets, the provision of reliable, environmentally robust and low cost customizable sensing devices is absolutely critical. Photonics technology is well suited for operation in industrial environments with increased electromagnetic noise and interference and a special class of low cost fiber-based sensors could provide a platform for such customizable solutions. The demanding problem of coolant fluid ageing has been selected and studied in order to design and implement efficient sensors for monitoring the ageing process as this could have a strong impact on manufacturing, environmental and occupational health issues related to coolant use. Furthermore the need to combinedly monitor coolants’ condition in remote places in an industrial infrastrucure establishes the requirements for wireless-enabled sensors able to operate in a wireless sensors network. Large core silica and polymer optical fibers have been identified as good candidate platforms for the development of sensors for pH monitoring as this was proved to be a parameter closely related to the rather complicated and multi-parametric ageing processes of such chemically complex fluids. The developed sensors demonstrated very good and reliable behavior for pH monitoring in a wide range from pH 3 to 11. However, their response in real coolants which have a quite complicated chemical composition and content nature further complicated by the developed biological load and a number of inorganic impurities, was quite different. The silica-based sensor, due probably to the better adhesion of the sol-gel derived sensitive overlayer, withstood the attack of the harsh coolant and operated quite reliably, being able to monitor the critical area of coolant degradation with a response of a few seconds. The simplicity of the proposed scheme together with the low power consumption characteristics allowed the integration of the sensing head in a ZigBee- based wireless node demonstrating the capability of this sensing platforms in a wider range of monitoring applications by employing these hybrid photonic-based wireless sensing nodes.

## Figures and Tables

**Table 1:** Chemical composition of industrial grade coolant fluids, Syntilo 81BF and Multan 61-3 DF.

Coolant	Material	Quantity (%)	Chemical Structure
Multan 61-3 DF (Henkel)	Polyalkylene glycol esters	N/A	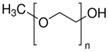
	Pyridine-2-thiol 1-oxide sodium salt	0.25–0.3	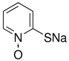
	Inorganic salts	N/A	N/A
Syntilo 81 BF (Castrol)	Triethanolamine	35–40	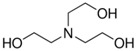
	Ethanolamine	1–5	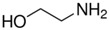
	3-Iodo-2-propynyl butylcarbamate	0.1–1	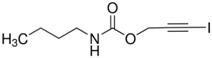
	Poly(quaternary ammonium chloride)	0.1–0.25	-

**Table 2:** pH indicators used for the development of the sol-gel derived pH sensitive overlayer.

Indicator	Chemical Structure	pH range
Bromophenol blue	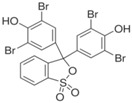	3.0–4.6
Chlorophenol red	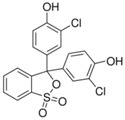	4.8–6.7
Cresol red	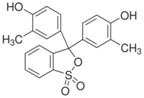	7.0–10.8
